# Osteoclast-derived exosomal miR-30a-3p promotes lead exposure-induced osteoporosis by triggering osteoblastic pyroptosis

**DOI:** 10.1042/CS20243438

**Published:** 2025-04-10

**Authors:** Yue Gao, Hang Zhang, Yinnong Jia, Yuanfang Chen, Luna Wang, Jie Ding, Wen Wang, Baoli Zhu, Liu Ouyang, Xu He, Yan An, Tingting Yu, Hengdong Zhang, Ming Xu

**Affiliations:** 1 Department of Occupational Disease Prevention, Jiangsu Provincial Center for Disease Control and Prevention, Nanjing 210009, China; 2 Jiangsu Province Engineering Research Center of Health Emergency, Nanjing 210009, China; 3 Gulou District Center for Disease Control and Prevention, Nanjing 210003, China; 4 Department of Pharmaceutical Sciences, School of Pharmaceutical Sciences, Kunming Medical University, Kunming 650500, China; 5 College of Modern biomedical industry, Kunming Medical University, Kunming 650500, China; 6 Yunnan Key Laboratory of Pharmacology for Natural Products, Kunming Medical University, Kunming 650500, China; 7 Engineering Research Center of Health Emergency, Jiangsu Provincial Center for Disease Control and Prevention, Nanjing 210009, China; 8 Taicang Center for Disease Control and Prevention, Suzhou 215400, China; 9 Department of Toxicology, School of Public Health, Suzhou Medical College, Soochow University, Key Laboratory of Geriatric Disease Prevention and Translational Medicine, Suzhou, Jiangsu 215123, China; 10 School of Public Health, Nanjing Medical University, Nanjing 211166, China; 11 Center for Global Health, School of Public Health, Nanjing Medical University, Nanjing 211166, China; 12 Department of Orthopaedics, Union Hospital, Huazhong University of Science and Technology, Wuhan 430022, China; 13 Department of Orthopaedics, Jilin City People’s Hospital, Jilin 132001, China; 14 Department of Medical Genetics, School of Basic Medical Sciences, Nanjing Medical University, Nanjing 211166, China

**Keywords:** exosomes, miR-30a-3p, osteoblasts, osteoclasts, Pb, pyroptosis

## Abstract

High lead (Pb) burden in humans disrupts bone homeostasis and can induce osteoporosis. Here, we report that osteoclast-derived exosomes (OC-Exos) were enriched in the plasma of patients with low bone mineral density and Pb exposure. Osteoclasts (OCs) secrete microRNA-enriched exosomes, through which miR-30a-3p is transferred to osteoblasts (OBs) to induce pyroptosis, leading to the aggravation of bone loss. Mechanistically, OC-Exo-packaged miR-30a-3p triggered pyroptosis in OBs by stimulating the NLRP3 inflammasome, activating the caspase-1 pathway, and up-regulating the expression of IL-1 and IL-18. Depletion of miR-30a-3p abolished the effects of OC-Exo and alleviated the symptoms of Pb-induced osteoporosis. Collectively, our results suggest that miR-30a-3p is highly expressed in exosomes derived from OCs and mediates OB pyroptosis, inhibiting bone formation through cellular communication in Pb-induced osteoporosis. Therefore, OC-Exo-packaged miR-30a-3p may be a novel risk factor for Pb-induced osteoporosis and holds prognostic value in evaluating bone formation.

## Introduction

Lead (Pb) burden has been reported in humans, animals, and plants [1]. As a common environmental pollutant, Pb tends to accumulate in the human body, particularly in bone tissue, leading to chronic toxicity [2,3]. Consequently, cumulative Pb burden causes the development of low bone mineral density (BMD) and ultimately triggers the onset of osteoporosis [4–6]. Osteoporosis is a global public health problem that affects approximately 319  million people, particularly adults over age 50 [7]. Normal bone remains in a delicate dynamic balance between osteoclastic bone resorption and osteoblastic bone formation. Pathologically, the disruption of this equilibrium results in diseases such as osteoporosis [8,9]. The association between Pb exposure and osteoporosis was observed in clinical examination and has been subsequently confirmed in animal models [2,10,11]. Studies have shown that Pb exposure leads to decreased body mass and BMD [12]. Li et al*.* reported that lumbar spine BMD decreased with an increase in blood lead levels (BLLs) in both children and adolescents [13]. Additionally, Khalil and Berlin indicated that the elevated BLL may directly contribute to the pathogenesis of osteoporosis [4,14]. A high concentration of Pb not only replaces Ca to affect crystallization but also affects bone remodeling by affecting the cellular activity of osteoblasts (OBs) and osteoclasts (OCs) [15]. However, the precise mechanisms underlying the pathogenesis of Pb-induced osteoporosis, especially the intercellular communication between OCs and OBs during this process, remain unclear.

Therefore, understanding the specific mechanisms of Pb-induced osteoporosis and identifying promising biomarkers are crucial to developing effective strategies to manage osteoporosis in patients exposed to environmental Pb. Osteoclastic bone resorption and osteoblastic bone formation are vital processes of bone homeostasis, occurring simultaneously and in co-ordination with each other [16]. As important messengers, exosomes have been studied extensively and have been shown to play a key role in cellular communication in several severe diseases; they may also participate in bone homeostasis [17]. Exosomes are cup-shaped bilayer membranes with a diameter ranging from 30 to 200 nm and are secreted as extracellular vesicles by all mammalian cells under physiological and pathological conditions [18,19]. By carrying microRNAs (miRNAs), proteins, and lipids with diverse functions, exosomes can systemically transport biological characteristics from parent cells to recipient cells. Therefore, understanding the pattern of exosome-exported cargo in disease is valuable to diagnostic and pathological studies [18,20]. In the context of bone disease, emerging evidence suggests that secreted miRNAs could serve as novel tools for investigating the pathological mechanisms and biomarkers of osteoporosis [21,22]. Li et al*.* reported that the transfer of OC-Exo-packaged miR-214-3p inhibited OB activity and bone formation in prednisone-induced osteoporosis [23]. However, in idiopathic osteoporosis, especially when induced by industrial and environmental Pb exposure, the corresponding OC-Exo-packaged miRNA expression profiles require further study.

Studies have demonstrated the involvement of extracellular vesicles in the regulation of osteoporosis. However, there is a lack of research investigating whether specific extracellular vesicles are involved in the regulation of Pb-induced osteoporosis. The results of our previous studies suggest that the aberrant expression of exosomal miR-30a-3p in OCs may be associated with the development of osteoporosis in response to Pb exposure. This indicates the potential of miR-30a-3p as a biomarker for diagnosing Pb-induced osteoporosis. However, the specific role and underlying mechanisms of miR-30a-3p in Pb-induced osteoporosis remain unclear, necessitating further investigation. Thus, we aimed to (1) identify miR-30a-3p exosomes as novel risk factors in Pb-induced osteoporosis and (2) elucidate the molecular function of miR-30a-3p exosomes and their content in cellular communication to gain a better understanding of Pb-induced osteoporosis.

## Materials and methods

### Participant characteristics

All participants received physical health examinations and had their BLL and BMD measured at the Jiangsu Provincial Center for Disease Prevention and Control between April and May 2021. Inclusion criteria for the research subjects were as follows: (1) BLL ≥ 400 μg/l, (2) *t*-values ≤ −1 SD, and (3) complete health monitoring files. The exclusion criteria were as follows: (1) diseases primarily involving bone metabolism, (2) a history of fracture within one year, (3) chronic treatment with drugs that affected normal bone metabolism such as calcium and hormones, and (4) poor compliance with BMD evaluation.

According to the inclusion and exclusion criteria and matching 1:1 for age and sex, six cases of Pb-exposed low-BMD (group A), six cases of Pb-exposed normal-BMD (group B), six cases with low BMD without Pb exposure (group C), and six healthy controls (group D) were enrolled in the testing set. In addition, 48 cases of Pb-exposed low-BMD, 50 cases of low-BMD, and 50 cases of healthy controls were enrolled in the validation set. The detailed clinical data are summarized in Table 1. The study was approved by the ethics committee of the Jiangsu Provincial Center for Disease Control and Prevention (JKJS2022-B002-01), in accordance with the principles of the Helsinki Declaration. Each participant signed a written informed consent before donating 5 ml of venous blood for further analysis.

**Table 1:** The clinical characteristics of participants

	Testing set					Validation set			
Variables	Group A (*n* = 6)	Group B (*n* = 6)	Group C (*n* = 6)	Group D (*n* = 6)	*P*	Group A (*n* = 48)	Group C (*n* = 50)	Group D (*n* = 50)	*P*
Sex (M/F)	(4/2)	(4/2)	(4/2)	(4/2)	1.000	(40/8)	(40/12)	(40/13)	0.507
Age (year)	43.33 ± 10.54	44.33 ± 5.79	45.00 ± 6.69	44.00 ± 5.18	0.983	46.92 ± 8.02	46.12 ± 7.43	44.66 ± 7.45	0.334
BMI (kg/m^2^)	23.02 ± 3.64	23.40 ± 2.46	23.00 ± 2.16	23.17 ± 1.21	0.992	24.32 ± 2.74	24.51 ± 2.73	23.89 ± 3.17	0.548
Smoking (yes/no)	(0/6)	(0/6)	(0/6)	(0/6)	1.000	(19/29)	(23/27)	(16/34)	0.357
Drinking (yes/no)	(0/6)	(0/6)	(0/6)	(0/6)	1.000	(18/30)	(21/28)	(12/38)	0.127
Work seniority/year	10.00 ± 4.77	10.63 ± 7.20	11.83 ± 7.22	9.5 ± 6.69	0.934	11.35 ± 5.79	11.02 ± 5.65	9.64 ± 5.13	0.266
Working hours/week	60.33 ± 12.09	57.17 ± 8.21	55.67 ± 5.47	52.33 ± 4.08	0.405	54.31 ± 7.06	52.50 ± 9.42	52.66 ± 12.49	0.610
BLL (μg/l)	525.13	474.55	32.75	26.35	0.002	510.65	51.75	54.4	0.000
	(467.35–585.08)	(440.43–512.93)	(28.65–48.05)	(20.00–34.88)		(447.55–510.65	(30.20–81.58)	(29.30–91.25)	

Age, BMI, work seniority, and hours were presented as mean value ± standard deviation, and BLL was presented as M (P25–P75).

BMI, body mass index. BLL, blood lead level.

### Cell lines

Murine RAW264.7 monocytes were purchased from the Cell Bank of the Chinese Academy of Sciences (Shanghai, China) and were maintained in high-glucose DMEM supplemented with 10% FBS, 100 U/ml penicillin, and 100 μg/ml streptomycin (Gibco, U.S.A.). MC3T3-E1 cell lines were gifted by the Central Laboratory of Suzhou University (Suzhou, China) and were cultured with αMEM supplemented with 10% FBS and 1% penicillin/streptomycin. All cell lines were cultured at 37°C in a humidified atmosphere (5% CO_2_, 95% air) and were negative for mycoplasma contamination.

### Animal model of osteoporosis

The animal experiments were conducted in compliance with the guidelines for ethical animal welfare, as approved by the Institutional Animal Care and Use Committee (IACUC) at Nanjing Medical University (IACUC-2106032). Forty Sprague-Dawley (SD) rats, eight weeks old and weighing 242.5 ± 11.05 g, were acquired from the Animal Core Facility of Nanjing Medical University (SCXK [SU] 2021–0001). Prior to the initiation of the experimental protocols, the rats were housed for a seven-day adaptation period in the designated facility. The rats were killed using CO_2_ gas in an enclosed chamber, followed by manual cervical dislocation. All experimental procedures involving animals were executed at the Animal Laboratory of Nanjing Medical University. A rat model of osteoporosis was then established by gavage of lead acetate (PbAc), as previously described [24]. Animals were randomly divided into five groups: the 2 mg/ml PbAc group (*n* = 8) (rats were treated with 2 mg PbAc/100 g body weight by oral gavage daily), the 4 mg/ml PbAc group (*n* = 8), the 8 mg/ml PbAc group (*n* = 8), the prednisone group (*n* = 8) (rats were administered prednisone solution at dose of 0.4 mg/100 g body weight by oral gavage daily), and the negative control (NC) group (*n* = 8) that received only deionized water. After the sequential exposure period (60 days) of the experiment, all rats were killed, and blood and bilateral femurs were collected. The left femur was isolated from the soft tissues for the measurement of micro-computed tomography (micro-CT). The right femur was obtained for bone histopathology.

### Blood specimen preparation

Venous blood samples in this study were collected and dispensed into an EDTA tube (5 ml), a heparin tube (2 ml), and a serum tube (3 ml). The blood sample in the EDTA tube was centrifuged at 3,000 rpm for 15 min at 4°C. The clear supernatant (plasma sample) was transferred to a labeled tube and stored at −80°C. The blood sample in the serum tube was centrifuged at 4,000 rpm for 10 min at 4°C after clotting for 30 min at room temperature. The clear yellow supernatant (serum sample) was collected and preserved at −80°C. The blood sample in the heparin tube was used for measurement of BLLs via atomic absorption spectrometry (AAS, ThermoFisher, U.S.A.).

### Immunohistochemical tartrate-resistant acid phosphatase/alkaline phosphatase staining

Tartrate-resistant acid phosphatase (TRAP)/alkaline phosphatase (ALP) double staining was applied to evaluate the activity of OCs, OBs, and the rat serum samples according to the manufacturers’ protocols. The right femurs of rats were isolated and fixed in 4% paraformaldehyde, demineralized in 20% ethylenediaminetetraacetic acid, and embedded in paraffin. Sagittal sections, 6 μm in thickness, were prepared from the center of the femur and stained. Image Pro Plus was used to measure and analyze the collected data image results. The number of TRAP- and ALP-positive cells was counted, and the area in the selected region was measured to calculate the number of OCs in the selected area/bone area.

### Preparation of OCs, TRAP staining, and TRAP activity assay

The receptor activator of NF-κB ligand (RANKL) and macrophage colony-stimulating factor (M-CSF) are two critical cytokines involved in the differentiation of OCs [25]. To investigate the effect of RANKL and M-CSF on OC differentiation, RAW264.7 cells were cultured in six-well plates for 24 h at a density of 2 × 10^4^ cells per well. After adherence to the wells with 90% confluency, the cells were cultured with RANKL (50  ng/ml) and M-CSF (50  ng/ml) differentiation-inducing medium for six days. The culture medium was replaced with fresh medium every other day. Cells were fixed with 4% formaldehyde and incubated with ethanol/acetone (50 v/50 v) for 1 min at −20°C and then incubated with a TRAP kit (Wako, Japan) for 30 min at 37°C. The multinuclear cells with TRAP-positive staining were recorded using a microscope (OLYMPUS IX71, Japan), and the cells containing three or more nuclei were identified as OCs. TRAP activity assay was performed according to the instructions of the TRAP kit (Beyotime, China). All experiments were repeated in triplicate with independent samples.

### Cytotoxicity assay

The effect of PbAc on cellular viability was assayed using the 3-(4,5-dimethylthiazol-2-yl)-2,5-diphenyltetrazolium bromide (MTT) assay. Briefly, 5 × 10^3^ OCs were seeded in 96-well plates and exposed to various concentrations of PbAc in a gradient of 0, 1, 5, 10, 50, or 100 μmol/l for 24 h. Then, 10  μl of MTT (Solarbio, China) was added and incubated for another 4  h at 37°C. Finally, 110  μl of Formazan was added and incubated for 10 min, and absorbance was measured at 490  nm using a microplate reader (ThermoFisher, U.S.A.).

### Exosome isolation

Exosomes were isolated from plasma or OCs using an exosome isolation kit (Invitrogen, U.S.A.) according to the manufacturer’s protocol. Briefly, supernatants collected from plasma or OCs were centrifuged at 10,000 rpm for 20  min to remove the cell debris. Then, 250 μl of PBS and 150 μl of the exosome precipitation reagent were added to 500 μl of the supernatant. The mixture was incubated at room temperature for 10  min and then centrifuged at 10,000  rpm for 5  min. Finally, the supernatant was discarded, and the tubes were centrifuged again at 10,000 rpm for 30 s. The resulting exosome pellet was resuspended in 200  μl of PBS and stored at −80°C.

### Transmission electron microscopy

For the identification of exosomes, the morphology of the extracted particles was observed using a transmission electron microscopy (TEM) (JEOL-1230, Japan). Briefly, freshly prepared exosomes were adsorbed onto a metal sample grid, fixed with 2.5% glutaraldehyde, and then stained using 10 μl uranyl-acetate saturated alcohol solution for 1 min. After blotting the excessive solution, the sample was dried and observed via electron microscopy at 80 kV.

### Nanoparticle tracking analysis

After diluting with PBS, exosome size distribution was measured by nanoparticle tracking analysis (NTA) using the NanoSight NS300 system (Malvern Instruments, U.K). The size distribution profile and particle concentration of exosomes were measured using NTA software.

### Western blot analysis

Following the different treatments, proteins from exosome samples or OCs were extracted using RIPA lysis buffer (Solarbio, China). Total protein concentration was determined using the bicinchoninic acid kit (Beyotime, China). The proteins were separated using 8–12% sodium dodecyl sulfate polyacrylamide gel electrophoresis (SDS-PAGE) and then transferred to polyvinylidene difluoride (PVDF) membranes. After adding QuickBlock™ blocking buffer (Beyotime, China) at room temperature for 15 min, the PVDF membranes were incubated overnight at 4°C with CD9 (ab92726, 1:2000), CD63 (ab217345, 1:2000), CD81 (ab109201, 1:2000), TSG101 (ab125011, 1:1000), TRAcP5 (ab191406, 1:1000), Sema 4D (ab231961, 1:1000), cathepsin K (CTSK) (ab187647, 1:1000), or GAPDH (ab181602, 1:1000) antibodies. The PVDF membranes were incubated with the corresponding HRP-conjugated secondary antibody (Beyotime, China) at room temperature for 1 h. Finally, the protein bands were visualized using ECL chemiluminescence reagent (Bio-Rad, U.S.A.).

### Extraction and sequencing of miRNA

Total miRNA was extracted from human plasma and OC-derived exosomes using the miRNeasy Micro Kit (Qiagen, Germany). The miRNA concentration was quantitated using a NanoDrop ND-100 Spectrophotometer (ThermoFisher, U.S.A.). The miRNA high-throughput sequencing was performed by CloudSeq Biotech (Shanghai, China). Individual libraries were constructed using 1 μg of total RNA isolated from each sample and then sequenced for 50 cycles on an Illumina NovaSeq 6000 sequencer, following the manufacturer’s instructions. Finally, the miRNAs with a fold change ≥2.0 and *P* ≤ 0.05 were selected as differentially expressed miRNAs.

### Quantitative RT-PCR

To evaluate miRNA expression, complementary DNA was reverse-transcribed according to the cDNA kit manufacturer’s instructions (GenePharma, China). Gene products were then amplified with an ABI-Prism 7500 Real-Time PCR System (Applied Biosystems, U.S.A.) using a SYBR Real-Time PCR kit (GenePharma, China). The relative level of miR-30a-3p was normalized to the expression of control U6 snRNA. Data were analyzed using the 2^−ΔΔCt^ method. Primer sequences are shown in Table 2.

**Table 2:** Primer sequences used for qRT-PCR

Gene		Primer sequence
U6	Forward	5′-CAGCACATATACTAAAATTGGAACG-3′
	Reverse	5′-ACGAATTTGCGTGTCATCC-3′
miR-30a-3p	Forward	5′-CGCCTTTCAGTCGGATGTT-3′
	Reverse	5′-CAGAGCAGGGTCCGAGGTA-3′
miR-103a-3p	Forward	5′-CAGATAGCAGCATTGTACAGGG-3′
	Reverse	5′-TATCGTTGTACTCCAGACCAAGAC-3′

### Micro-CT

All femurs were scanned using the Hiscan XM Micro-CT (Hiscan, China) in the sagittal plane, transverse plane, and coronal plane. The X-ray tube settings were 80 kV and 100 μA, and images were acquired at a resolution of 25 μm. A 0.5° rotation through a 360° angular range with 50-ms exposure per step was applied. HiScan software was used for 3D model reconstructions and bone histomorphometry parameter analysis, including bone volume fraction (bone volume/total volume, BV/TV), trabecular thickness (Tb.Th), trabecular number (Tb.N), trabecular separation (Tb.Sp), and BMD. Data were obtained at a region of interest that began 1.5 mm distance from the growth plate and extended 2.0  mm distally.

### Transfection models

siRNA or miRNA was transfected into cells using the Xfect RNA Transfection Reagent (Takara, Japan) according to the manufacturer’s instructions. FAM-tagged miR-30a-3p mimic (GenePharma, China), miR-30a-3p inhibitor (GenePharma, China), and siNF-κB1 (Santa, U.S.A.) were added at a transfection concentration of 20 μM.

### Co-culture and transfection experiments

The OB precursor cells (MC3T3-E1) were seeded in a six-well plate at a density of 2 × 10^5^ cells per well and differentiated into OBs with the osteogenic α-MEM. The RAW264.7 cells were seeded in six-well plates and induced into OCs using complete DMEM containing RANKL and M-CSF. After differentiation, OCs were pretreated with 5 μM PbAc for 24 h and washed with PBS, and then co-cultured with OBs using a transwell system for 24 h. All co-culture experiments were performed in the exosome-free F/12 medium. After co-culture, RNaseA, TritonX-100, GW4869, or DMSO (as a solvent control) was added, followed by miRNA expression analysis.

### Cell viability assay

CCK8 assay was performed to evaluate OB cell viability. After OCs were co-cultured with OBs for 24 h, 1 ml medium and 100 μl of CCK8 reagent were added to each well, and they were incubated for 2  h at 37°C. Finally, the absorbance was measured at 450  nm using a microplate reader (ThermoFisher, U.S.A.).

### Lactate dehydrogenase assay

To determine cytotoxicity, the lactate dehydrogenase (LDH) assay kit (Nanjing Jiancheng Biology Engineering Institute, China) was used to measure LDH released from OBs. Absorbance was measured at 450  nm using a microplate reader (ThermoFisher, U.S.A.).

### Hoechst 33342/propidium iodide fluorescence staining

MC3T3-E1 cells were double-stained with Hoechst 33,342 and propidium iodide (PI) to assess pyroptosis. The cells were cultured in a six-well plate at a density of 2 × 10^5^ cells/well and co-cultured with Pb-treated OCs in a transwell chamber or pretreated with siRNA for 24 h. The cells in each group were washed three times with PBS and then stained with 2.5 μl Hoechst 33,342 and 2.5 μl PI (Beyotime, China) for 30 min in the dark. Then, images were taken at 20× magnification under a fluorescence microscope (OLYMPUS IX71, Japan).

### Exosome labeling

The fluorescent dye 1,1'-dioctadecyl-3,3,3',3'-tetramethylindocarbocyanine perchlorate (DiI) was purchased from Beyotime (China) and was used to label exosomes. Purified exosomes were incubated with DiI (10 μM) for 20 min at 37°C and then ultracentrifuged at 120,000 × g for 90 min to remove the free dye. After washing twice in PBS, the labeled exosomes were resuspended in PBS prior to use.

### Bone-formation assay

A total of 36 SD rats (7–8  weeks old, female vs. male 1:1) were randomly divided into the following four groups: NC-Exo, OC-Ex, Pb-OC-Exo, and (Anti-miR-30a-3p)-OC-Exo groups. To monitor the bone-targeting properties of the exosomes derived from OCs, DiI-labeled NC-Exo and OC-Exo were monitored using a fluorescence imaging system (IVIS imaging system, U.S.A.) with excitation and emission wavelengths of 549 and 565 nm, respectively. Then, 0.5 ml of DiI-labeled NC-Exo or OC-Exo was administered via tail vein injection. At 12  h after injection, the rats were sacrificed and biphotonic imaging was carried out. To assess the effect of OC-Exo-packaged miR-30a-3p on the occurrence of osteoporosis, exosomes (210 μg per rat) isolated from NC, OCs, Pb-treated OCs, or (Anti-miR-30a-3*P*+Pb)-treated OCs were injected into rats every other day for 30 days; these rats were designated as the NC-Exo, OC-Exo, Pb-OC-Exo, and (Anti-miR-30a-3*P*+Pb)-OC-Exo groups, respectively. Then, micro-CT was used to observe the femur microarchitecture and to obtain the bone histomorphometry parameters of each group.

### Luciferase reporter assays

The online databases TargetScan (www.targetscan.org) and miRDB (mirdb.org/miRDB) were used to predict the target genes of miR-30a-3p. NF-κB1 and IKBKG wildtype. Then, mutant fragments containing miR-30a-3p binding sites were designed, synthesized, and inserted into the dual luciferase reporter vector pmirGLO. Mimics were co-transfected into MC3T3 cells and received after 48 h . The cells were collected, and dual luciferase activity was detected using a luciferase assay kit. The ratio of firefly luciferase activity to renilla luciferase activity was used to calculate relative luciferase activity.

### Statistical analysis

All statistical analyses were performed with SPSS 26.0 software (version 26.0 for mac, U.S.A.) and Graphpad Prism software (version 9 for mac, U.S.A.). The data are expressed as mean ± standard deviation (SD), unless noted otherwise, and one-way analysis of variance was used to compare statistical differences among groups. The Chi-square test was used to determine the differences in categorical variables, and the Wilcoxon rank-sum test was used for non-normal data. The differences with *P* < 0.05 were considered statistically significant.

## Results

### Clinical characteristics of Pb-exposed and non-Pb-exposed populations

The clinical characteristics and grouping information of the participants included in the testing set and validation set are summarized in Table 1. In both sets, the plasma samples were collected and exosome sequencing was performed. As expected, no statistically significant difference was observed between the groups in the testing and validation sets regarding age, body mass index (BMI), smoking, drinking, work seniority, or work hours. However, BLLs were remarkably different between the groups in both the testing (*P* < 0.005) and validation sets (*P* < 0.001).

### Characterization and source identification of exosomes secreted by Pb stimulation

**Figure 1:**
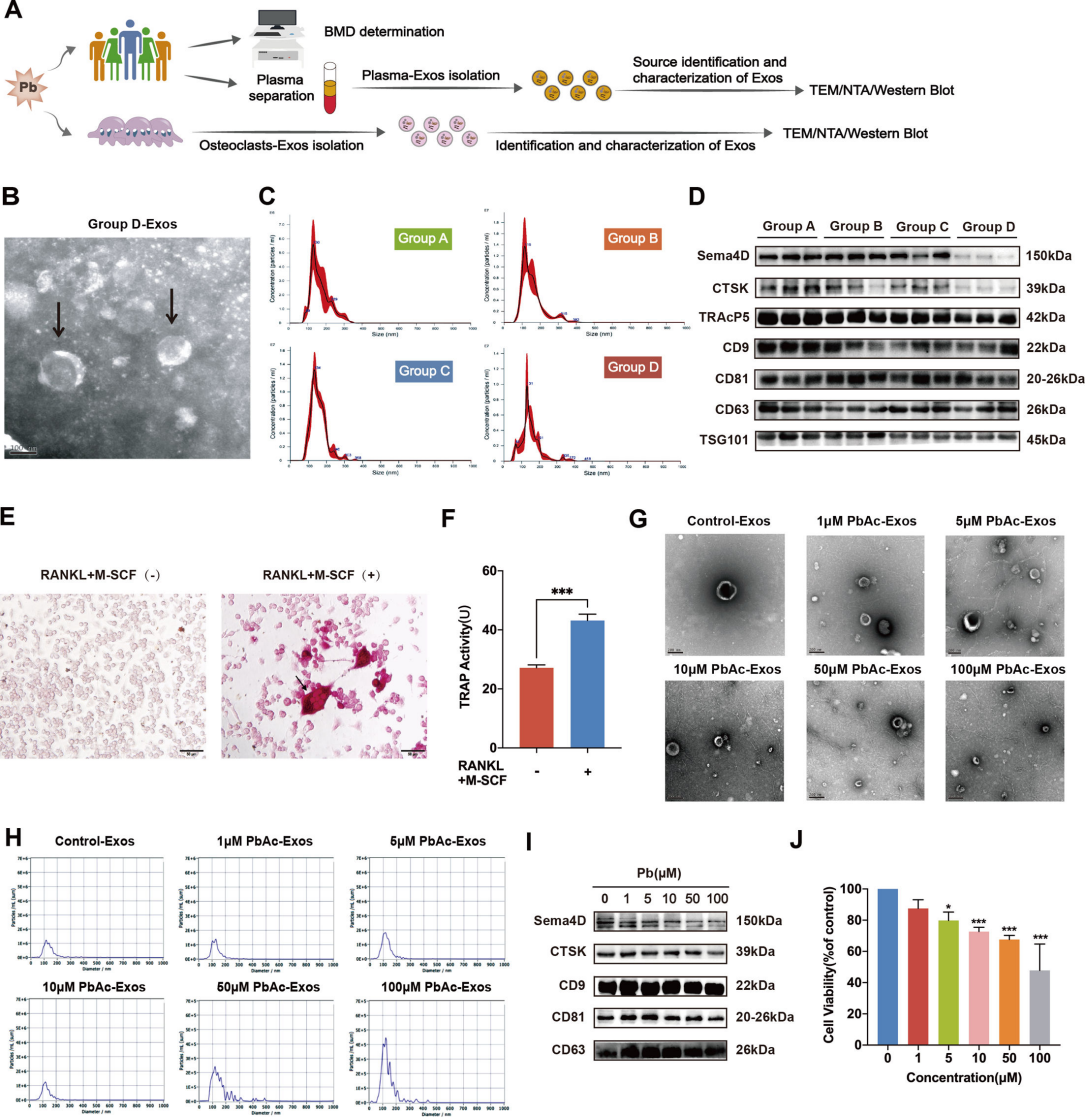
Identification and characterization of exosomes derived from human plasma and OCs after lead (Pb) exposure. **(A**) Schematic diagram of designing population and osteoclast. Plasma and bone mineral density were collected from individuals with or without Pb exposure. Patients were divided into Pb-exposed low-BMD (group A), Pb-exposed normal-BMD (group B), low-BMD without Pb exposure (group C) and healthy control (group D) group, according to the BLL and BMD. (**B**) Visualization of purified plasma-Exos from the healthy control group. The shape of plasma-Exos was confirmed by TEM identification. Scale bar, 100 nm. (**C**) The size distributions of group A/group B/group C/group D-Exos were identified by NTA. The X-axis represents exosome size, and the Y-axis represents the relative concentration of exosomes. (**D**) Western blot analysis of the OC-marker proteins (Sema4D, CTSK, and TRAcP5) in the plasma-Exos isolated from low-BMD patients with or without Pb exposure and healthy volunteers with or without Pb exposure, and the Exos-specific proteins CD9, CD63, CD81, and TSG101 in plasma-Exos were determined. (**E**) Representative images of TRAP staining of RAW 264.7 cells with and without M-CSF (50  ng/ml) and RANKL (50  ng/ml) treatment. Multinucleated OCs (≥3 nuclei) with TRAP-positive staining (red color) were counted as mature OCs. Scale bar, 50 μm. (**F**) RAW 264.7 cells were cultured in the absence or the presence of M-CSF and RANKL. The relative expression of the TRAP enzyme was detected using the kit. (**G**) Purified OC-Exos treated with various concentrations of PbAc were identified using TEM. Scale bar, 200 nm. (**H**) NTA results suggested that OC-Exos treated with various concentrations of PbAc were about 50–200 nm in diameter. (**I**) The OC-marker proteins (Sema4D and CTSK) and the Exo-specific proteins (CD9, CD63, and CD81) were detected in OC-Exos treated with various concentrations of PbAc. (**J**) The viability of OCs treated with various concentrations of PbAc was measured by MTT. **P < 0.01 and ***P < 0.001. BMD, bone mineral density; BLL, blood lead level; CTSK, cathepsin K; M-CSF, recombinant mouse SCF protein; NTA, nanoparticle tracking analysis; OCs, osteoclasts; PbAc, lead acetate; RANKL, recombinant mouse TRANCE/RANKL 11 protein; TEM, transmission electron microscopy; TRAP, tartrate-resistant acid phosphatase.

To verify the existence of exosomes in Pb-induced osteoporosis and their involvement in the pathogenesis of Pb-induced osteoporosis, we identified exosomal miRNAs that might participate in Pb-induced osteoporosis. Four groups were established, including low-BMD patients with or without Pb exposure (group A or C) and healthy volunteers with or without Pb exposure (group B or D). Plasma samples were then collected, and exosomes were isolated (plasma-Exo) (Figure 1A). The presence of plasma-Exos was confirmed by TEM, showing round vesicles with a double-layer membrane (Figure 1B). The NTA results from the plasma-Exos indicated that the diameter of isolated vesicles in the four groups was mainly 150–170  nm, which is consistent with the typical size of exosomes (Figure 1C). The presence of the exosome-specific proteins CD9, CD63, CD81, and TSG101 was confirmed in plasma-Exos by Western blotting (WB) (Figure 1D). Additionally, the expression of the osteoclastic marker proteins (semaphorin 4D [Sema4D], CTSK, and TRAcP5) in plasma-Exos was evaluated (Figure 1D). The results showed that, compared with the healthy controls (group D), the expression of OC markers Sema4D and CTSK was higher in Pb-exposed low-BMD patients (group A) and Pb-exposed normal-BMD patients (group B), indicating that Pb exposure may promote the high expression of OC markers in exosomes. The expression of TRAcP5 was higher in Pb-exposed low-BMD patients (group A) than in healthy controls (group D), which is consistent with the expectation that Pb exposure increases the release of OC-derived exosomes (OC-Exos). This further supports the hypothesis that Pb exposure may facilitate the release of OC-Exos.

In the Pb-induced osteoporosis cell model, we also observed the involvement of extracellular vesicle-derived miRNAs in the pathogenic mechanism of Pb. TRAP staining images revealed that a higher number of RAW 264.7 cells differentiated into TRAP-positive OCs when stimulated with RANKL and M-CSF compared with those without RANKL and M-CSF treatment (Figure 1E). Consistently, TRAP levels were significantly higher in the RAW 264.7 cells stimulated with RANKL and M-CSF than in the control group (Figure 1F), demonstrating the successful generation of OCs. After incubating differentiated OCs with various concentrations of PbAc, the effect of Pb on OC-Exos was evaluated. TEM confirmed that OC-Exos exhibited the typical characterization of exosomes, with a diameter of 100 nm and round morphology (Figure 1G). NTA revealed that the isolated OC-Exos from the control group had a peak diameter of 116 nm. Similarly, the peak diameter of OC-Exos isolated from the 5-μM PbAc-treated group was 112 nm, and the mean sizes of the OC-Exos in the control and 5-μM PbAc groups were comparable (Figure 1H). Further, the osteoclastic marker proteins CTSK and Sema4D were positive in OC-Exos treated with PbAc (Figure 1I). Interestingly, the expression of the transmembrane proteins CD81 and CD63 was initially increased and then decreased with increasing PbAc concentration (Figure 1I). At the concentration of 5 μM PbAc, the exosomal membranes remained intact, with a regular diameter. However, at PbAc concentrations above 10 μM, the membrane appeared to be damaged. The exosomal proteins decreased due to decreased OCs and a reduction in secreted exosomes. In addition, OC cell viability was significantly decreased in a concentration-dependent manner with PbAc treatment, compared with the control group (Figure 1J). As described above, the expression of exosomal marker proteins was significantly higher in the 5-μM PbAc group than in the control group (*P* < 0.001), and the cell viability of this group remained at approximately 80%. Therefore, a concentration of 5 μM PbAc was selected as the optimal concentration for subsequent experiments.

### OC-Exo-packaged miR-30a-3p was highly correlated with Pb exposure in patients with osteoporosis

**Figure 2:**
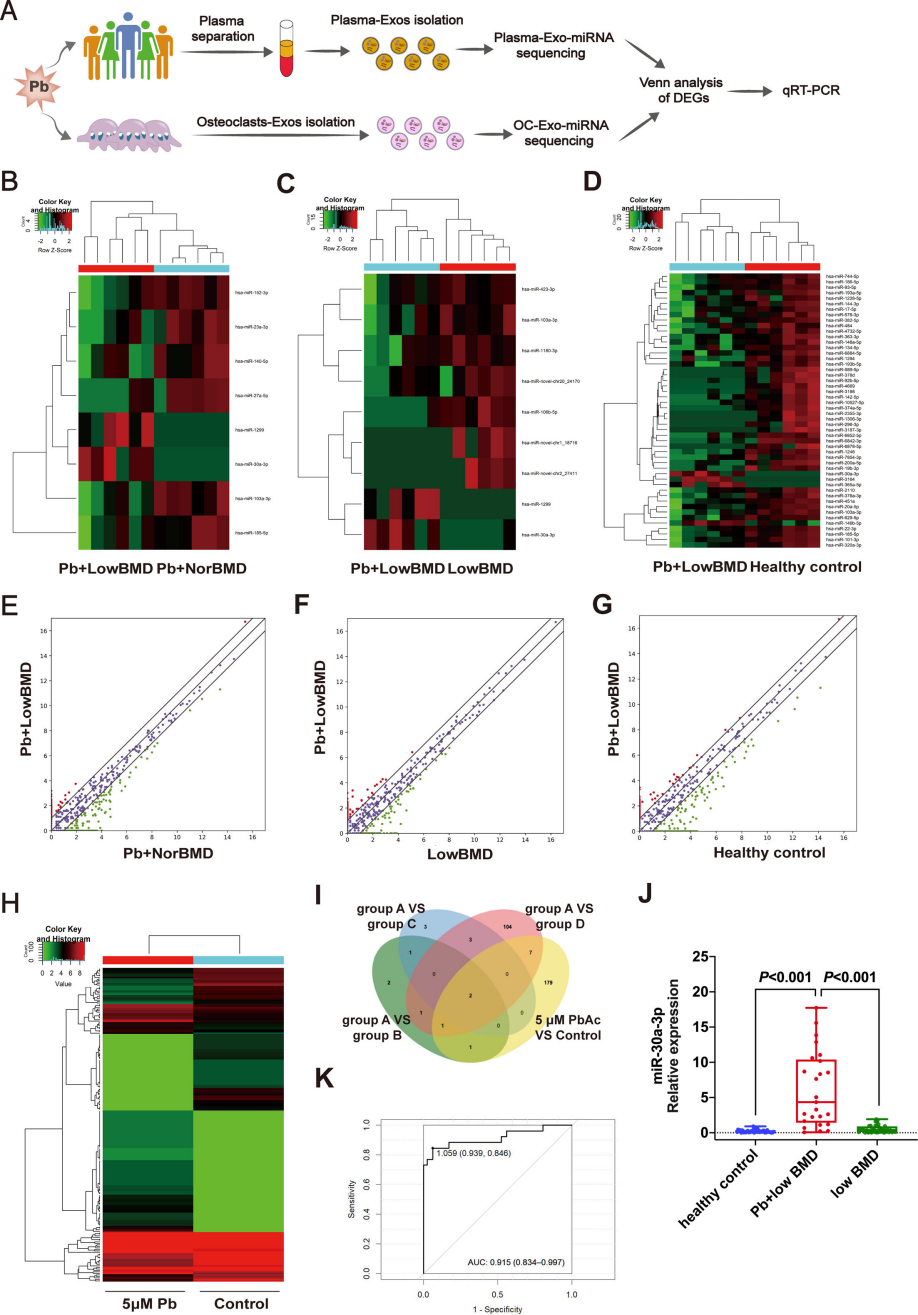
Profiling of miRNAs in plasma/OC-Exos and selection of exosomal miR-30a-3p. **(A**) Schematic diagram of the sequencing design of plasma-Exos and OC-Exos. Plasma-Exos from Pb-exposed low-BMD (group A), Pb-exposed normal BMD (group B), low BMD (group C), and healthy control (group D) groups and 5-μM PbAc OC-Exos were collected for sequencing. (**B**) Heatmap of differentially expressed plasma-Exo-miRNAs in groups A and B. Red and green indicate high expression levels and low expression levels, respectively. (**C**) Heatmap of differentially expressed plasma-Exo-miRNAs in groups A and C. Red and green indicate high expression levels and low expression levels, respectively. (**D**) Partial heatmap of differentially expressed plasma-Exo-miRNAs in groups A and D. Red and green indicate high expression levels and low expression levels, respectively. (**E**) Volcano plots of plasma-Exo-miRNAs in groups A and B. The red and green plots indicate the differentially expressed plasma-Exo-miRNAs. (**F**) Volcano plots of plasma-Exo-miRNAs in groups A and C. The red and green plots indicate the differentially expressed plasma-Exo-miRNAs. (**G**) Volcano plots of plasma-Exo-miRNAs in groups A and D. The red and green plots indicate the differentially expressed plasma-Exo-miRNAs. (**H**) Partial heatmap of differentially expressed OC-Exo-miRNAs in 5 μM PbAc and control. Red and green indicate high expression levels and low expression levels, respectively. (**I**) Venn diagram showing the overlapping numbers of differentially expressed miRNAs in the plasma-Exos and OC-Exos. (**J**) Real-time PCR analysis of the differential expression of miR-30a-3p in plasma-Exos between Pb-exposed low-BMD, Pb-exposed normal-BMD, and healthy control groups. U6 small nuclear RNA was used as the internal control. (**K**) ROC curve was used to analyze the diagnostic ability of miR-30a-3p in Pb-induced osteoporosis patients. The true-positive rate and the false-positive rate of ROC curve were calculated using the true-positive rate and the false-positive rate as the horizontal and vertical co-ordinates, respectively. BMD, bone mineral density; OCs, osteoclasts; PbAc, lead acetate.

To identify the differential expression profile in Pb-exposed low-BMD patients, plasma-Exo-miRNA extracted from patients from the testing sets was analyzed by miRNA sequencing and qRT-PCR (Figure 2A). The clinical information of the recruited patients is shown in Table 1. Compared with the Pb-exposed normal BMD group, two up-regulated miRNAs and six down-regulated miRNAs were found in the plasma exosomes of Pb-exposed low-BMD patients (Figure 2B and E). Moreover, two up-regulated and seven down-regulated miRNAs were identified in the plasma exosomes of Pb-exposed low-BMD patients compared with the low BMD group without Pb exposure (Figure 2C and F). We also identified four up-regulated miRNAs and 119 down-regulated miRNAs in the plasma exosomes of Pb-exposed low-BMD patients compared with healthy controls (Figure 2D and G). Furthermore, we obtained sequencing results of OC-Exo-miRNA in a cellular model to perform cross-comparison with the results from the four groups and to identify common exosomal miRNAs. Results showed that 190 miRNAs were differentially expressed between the 5-μM PbAc-treated group and the untreated controls, among which, 105 miRNAs were significantly increased and 85 miRNAs were decreased (Figure 2H). Finally, based on the above sequencing results, intersection analysis and Venn diagram analysis were performed. Two co-differentially expressed miRNAs were identified, and the up-regulated miR-30a-3p was selected for further validation (Figure 2I). The selection of miR-30a-3p was based on its well-documented role in bone metabolism and its involvement in pathways related to oxidative stress and mitochondrial function, which are critical in Pb-induced cellular damage [26–28]. In contrast, miR-103a-3p is widely recognized as a biomarker in various cancers, such as osteosarcoma [29], colorectal cancer [30], and glioma [31], in which its expression is significantly altered. Its broad involvement in multiple tumor types could lead to potential confounding effects in interpreting our results. Therefore, to ensure the specificity and clarity of our findings, we prioritized miR-30a-3p for validation in this study. The presence of miR-30a-3p was verified by qRT-PCR using plasma-Exos from the patients in the validation set. Consistently, miR-30a-3p was found to be significantly up-regulated (*P* < 0.001) in Pb-exposed low-BMD patients compared with low-BMD patients without Pb exposure (positive control) and healthy controls (Figure 2J). We further analyzed the efficacy of peripheral blood miR-30a-3p expression in the diagnosis of Pb-induced osteoporosis. The ROC curve for miR-30a-3p had an AUC value of 0.915, with an optimal cutoff value of 1.059, and the predicted sensitivity and specificity were 93.9% and 84.6%, respectively (Figure 2K). These results indicate that miR-30a-3p might serve as a metal exposure biomarker in low-BMD patients with Pb exposure.

### Pb exposure induced osteoporosis and up-regulated OC-Exo-packaged miR-30a-3p *in vivo*

**Figure 3:**
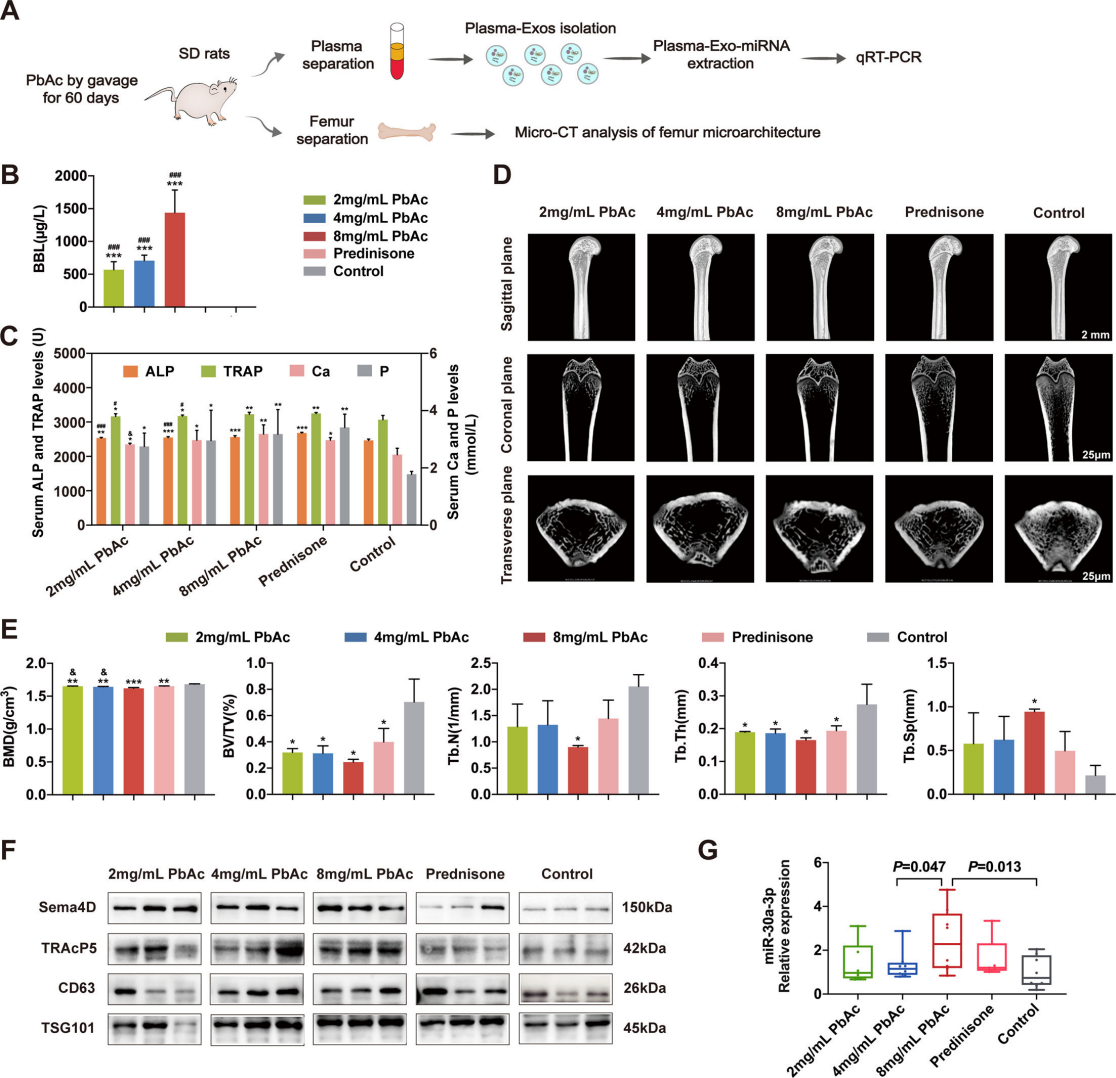
Reduced bone formation and elevated plasma exosomal miR-30a-3p in SD rats after treating with PbAc. (**A**) Schematic diagram illustrating the experimental design. Plasma and bone samples were collected after the SD rats were gavaged with 2 mg/ml PbAc, 4 mg/ml PbAc, 8 mg/ml PbAc, 0.4 mg/ml prednisone, and deionized water for 60 days, *n* = 8 mice per group. (**B**) Blood lead levels of rats in each group were measured using atomic absorption spectrometry. (**C**) The concentration of TRAP, ALP, Ca, and P in the blood of rats in each group. (**D**) Sagittal, coronal, and transverse views of the trabecular architecture of rat femurs in each group were analyzed by micro-CT. Scale bar, 25 μm. (**E**) Micro-CT microstructure analysis of femoral trabeculae in each group after feeding by gavage for 60 days. (**F**) The OC-marker proteins (Sema4D and TRAcP5) and the Exos-specific proteins (CD63 and TSG101) were evaluated using Western blotting. (**G**) The level of miR-30a-3p in plasma-Exos was determined using real-time PCR. U6 small nuclear RNA was used as the internal control. *P < 0.05, **P < 0.01, and *** *P* < 0.001 compared with the control group. ^#^*P*<0.05, ^##^*P*<0.001, and ^###^*P*<0.001 compared with Prednisone group. ^&^*P*<0.05 compared with 8 mg/ml PbAc group. BMD, bone mineral density; BV/TV, bone volume/total volume; Tb.N, trabecular number; Tb.Th, trabecular thickness; Tb.Sp, trabecular separation.

To explore the performance of miR-30a-3p in plasma-Exos *in vivo*, we established a Pb-induced osteoporosis rat model (Figure 3A). The BLLs were determined by atomic absorption spectrophotometry, as shown in Figure 3B. The amount of Pb was gradually elevated with increased dosages of PbAc in each exposure group. The amount of Pb was significantly higher in the exposure groups (2, 4, and 8 mg/ml PbAc treatment groups) than in the control and prednisone groups, where it was undetectable (*P* < 0.001). In addition, the levels of the bone resorption marker TRAP and the bone formation marker ALP were significantly higher (*P* < 0.05) in PbAc-treated rats than in controls. However, these markers were significantly lower (*P* < 0.05) in 2 mg/ml and 4 mg/ml PbAc-treated rats than in the prednisone group, and there was no difference in TRAP level in 8 mg/ml PbAc-treated rats (Figure 3C). Similarly, the concentrations of Ca^2+^ and P^3+^ were higher in PbAc-treated rats than in controls.

Furthermore, micro-CT scanning of femurs revealed a poorly organized trabecular architecture, weak intertrabecular connectivity, and low bone mass in the PbAc and prednisone-treated rats compared with the control group (Figure 3D). Consistently, the micro-CT quantitative analysis confirmed a decrease in BMD, BV/TV, Tb.Th, and Tb.N, as well as an increase in Tb.Sp in the 8 mg/ml PbAc-treated rats compared with the control group. We also observed a concentration-dependent decrease in BMD caused by PbAc (Figure 3E). These results demonstrated that the 8 mg/ml PbAc-treated rat femurs were substantially different from the control rats, confirming the successful establishment of the osteoporotic rat model via Pb treatment.

Subsequently, we isolated exosomes from the peripheral blood plasma of rats and evaluated the levels of exosomal markers (CD63 and TSG10) and osteoclastic markers (Sema4D and TRAcP5). As shown in Figure 3F, rat plasma-Exos were positive for CD63 and TSG101 proteins. Compared with non-treated and prednisone‐treated rats, PbAc-treated rats demonstrated higher expression of the Sema4D and TRAcP5 proteins. Additionally, PbAc increased the expression of exosomal markers and osteoclastic markers in a dose-dependent manner. The increase in exosomal proteins was coincident with an increase in osteoclastic marker proteins. Next, total miRNA was extracted from the plasma exosomes of rats, and the miR-30a-3p level was measured. The qRT-PCR results were consistent with the miRNA sequencing outcome, demonstrating a significant up-regulation of miR-30a-3p in the OC-Exos (*P* = 0.013) in the plasma of PbAc‐treated rats compared with the NC (Figure 3G). These data suggested that OC-Exos were released into the circulation in rat models with Pb-induced osteoporosis, and the up-regulation of miR-30a-3p was detected in the plasma of osteoporotic rats, consistent with previous results in both patient-Exos and OC-Exos.

### OC-Exos targeted bone and OC-Exo-miR-30a-3p inhibited bone formation *in vivo*

**Figure 4:**
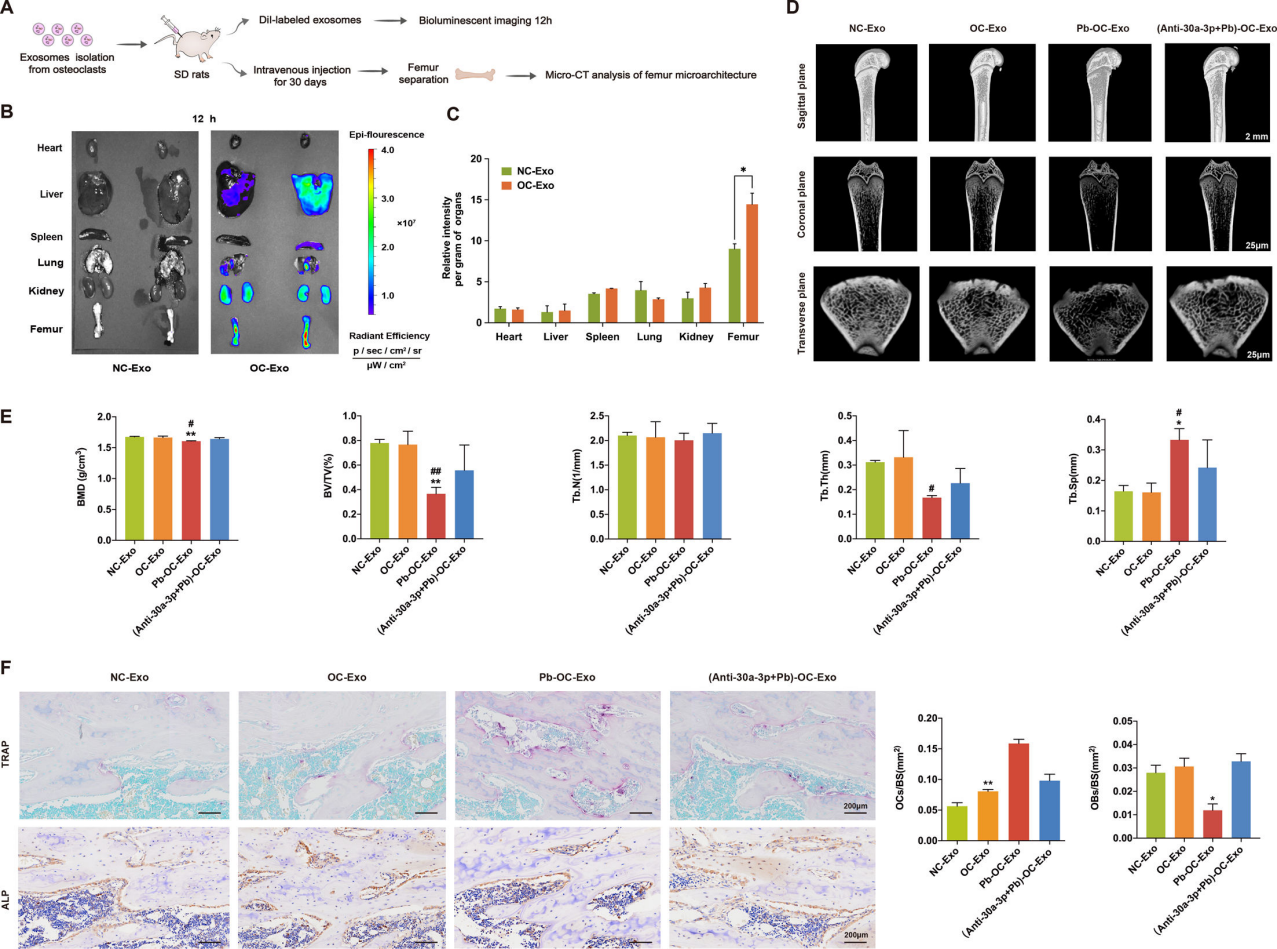
The osteoclast (OC)-derived exosomal miR-30a-3p secreted from Pb-treated OCs inhibits bone formation *in vivo*. Exosomes isolated from NC/OCs/Pb-treated osteoclasts/(Anti-miR-30a-3*P*+Pb)-treated OCs were intravenously injected into SD rats, which were designated as NC-Exo, OC-Exo, Pb-OC-Exo, and (Anti-miR-30a-3*P*+Pb)-OC-Exo, respectively. (**A**) Schematic diagram showing the process of establishing the rat model of exosomal miR-30a-3p, *n* = 6 mice per group. (**B**) Representative bioluminescent images of organs dissected from rats that were administered PBS (NC-Exo) or DiI-labeled OC-exosomes (OC-Exo). Organs were dissected 12 h after administration. Luminescence intensity ranges from low (blue) to high (red). (**C**) Quantitative analysis showed the relative fluorescence intensities per gram of different organs. (**D**) Representative micro-CT images of the femur from the rats administered NC-Exo, OC-Exo, Pb-OC-Exo, or (Anti-miR-30a-3*P*+Pb)-OC-Exo. Scale bar, 25 μm. (**E**) Values of micro-CT parameters (BMD, BV/TV, Tb.N, Tb.Th, and Tb.Sp) at the femur from the rats administered NC-Exo, OC-Exo, Pb-OC-Exo, or (Anti-miR-30a-3*P*+Pb)-OC-Exo. Values are means ± SEM. *P < 0.05, **P < 0.01, and ***P < 0.001. (**F**) Rat femur TRAP/ALP staining and quantitative analysis, scale bar, 200 μm, *P < 0.05,**P < 0.01. OBS/BS, osteoblast surface/bone surface area ratio; OCS/BS, ratio of osteoclast surface area/bone surface area; SD, Sprague-Dawley.

To detect *in vivo* bone-targeting effects, exosomes isolated from the supernatant of OCs (OC-Exos) or an equal amount of PBS (NC-Exo) were labeled with DiI and injected intravenously into rats for imaging. At 12-h post-injection, the intraosseous fluorescence signal was detectable, while no fluorescence was observed in the NC-Exo group (Figure 4B). The biodistribution of exosomes demonstrated that OC-Exos predominantly accumulated in the femur (Figure 4C).

Further, exosomes isolated from the supernatant of the NC, OC, Pb-treated OC, and (Anti-miR-30a-3*P*+Pb)-treated OC groups were injected intravenously for 30 consecutive days. In the micro-CT analysis of the femurs, typical morphologies such as the osteopenia, destructive trabecular structure, and increased trabecular space were observed in the rats treated with Pb-OC-Exo (Figure 4D). Quantitatively, the BMD, BV/TV, and Tb.Th were lower in rats treated with Pb-OC-Exo than in NC-Exo- and OC-Exo-treated rats (Figure 4E). We found that trabecular bone mass and trabecular destruction in rats treated with (Anti-miR-30a-3*P*+Pb)-Exo exhibited no statistically significant differences compared with NC-Exo rats, as verified by parameters including BMD, BV/TV, Tb.Th, Tb.N, and Tb.Sp (Figure 4E). Finally, we stained the bone tissue with Trap/ALP, and the Trap staining showed that the Trap enzyme in the OCs was dyed purple red. The number of OCs in the rat femurs increased after administration of Pb-OC-Exo and was significantly higher than that in the Anti-30-Pb-OC-Exo group (*P* < 0.05). ALP staining showed that the ALP enzyme in the OBs of rat femurs was stained brown. The number of OBs in rat femurs was decreased after administration of Pb-OB-Exo and was lower than that in the PBS, OB-Exo, and Anti-30-Pb-OB-Exo groups (*P* < 0.05) (Figure 4F). These data suggested that OC-Exo-miR-30a-3p produced by Pb exposure inhibited bone formation *in vivo*, and the administration of the miR-30a-3p inhibitor in OCs restored bone formation.

### Pb-treated OCs secreted Exo-packaged miR-30a-3p and induced pyroptosis in OBs

**Figure 5:**
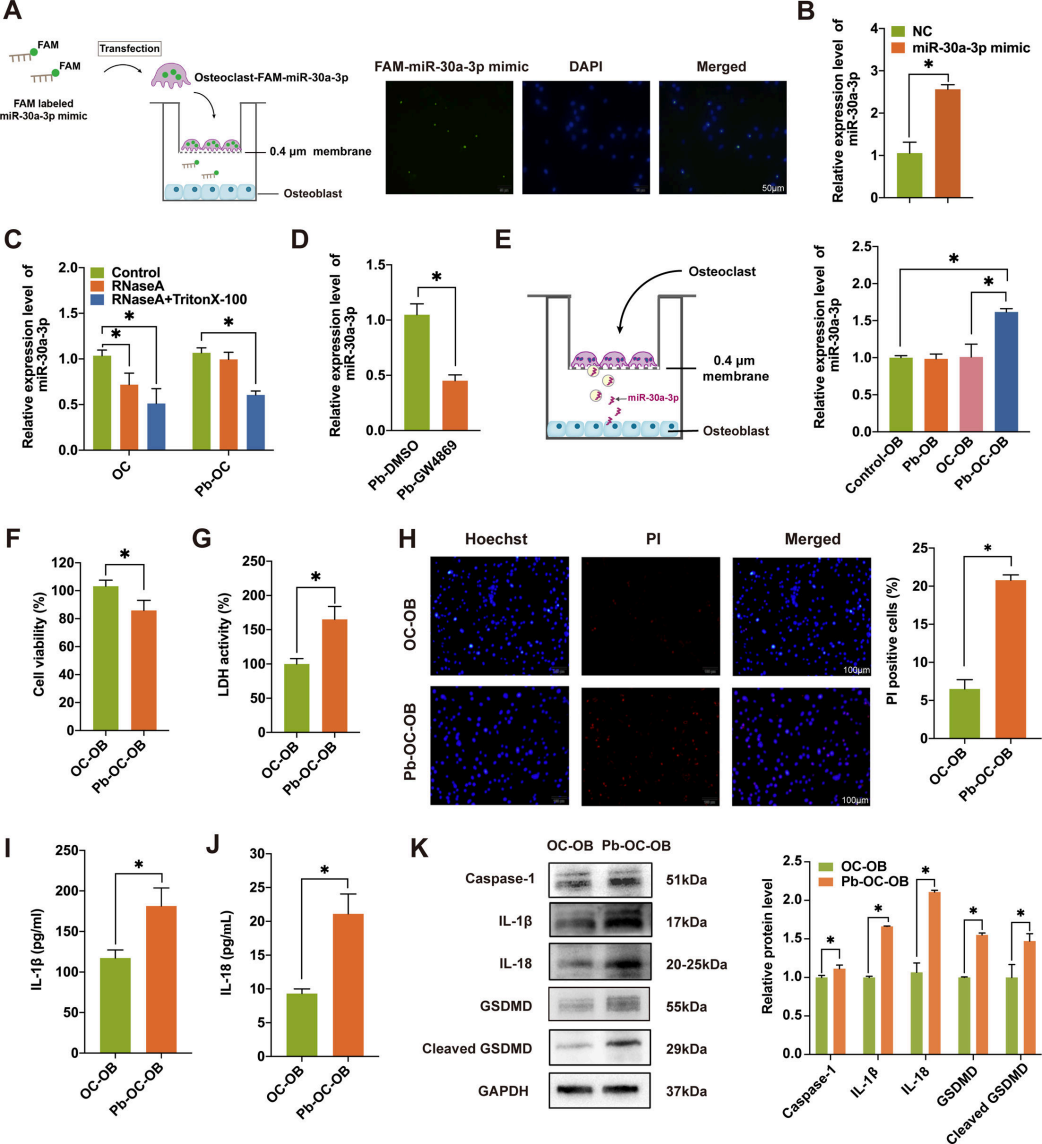
Pb-treated OCs secrete Exo-packaged miR-30a-3p and induce pyroptosis in OBs. (**A**) Scheme of experiment. OCs were transfected with the FAM-tagged miR-30a-3p mimic to generate OCs-FAM-miR-30a-3p and were co-cultured with OBs in a 0.4-μm transwell plate. The transfection was confirmed by confocal microscopy. Scale bar, 50 μm. (**B**) Expression of miR-30a-3p in OBs after incubation with FAM-miR-30a-3p mimic transfected or NC-OCs. (**C**) The level of miR-30a-3p in the culture medium of non/Pb-treated OCs incubated with 2 μg/ml RNaseA alone or in combination with 0.1% Triton X-100 was determined using RT-qPCR. (**D**) Expression of miR-30a-3p in the culture medium of Pb-treated OCs added with DMSO (as a control) and culture medium of Pb-treatedGW4869 (exosome inhibitor) was measured. (**E**) Left panel: Schematic illustration of OBs co-cultured with OCs. Right panel: Level of miR-30a-3p in OBs after Pb treatment or incubation with non/Pb-treated OCs. (**F**) The viability of OBs after incubation with non/Pb-treated OCs was evaluated using a CCK-8 assay. (**G**) The LDH released from OBs after co-culture. (**H**) Photomicrograph images of double-fluorescence staining with PI (red) and Hoechst 33,342 (blue). Scale bar, 100 μm. (**I**) After incubation with non/Pb-treated OCs for 24 h, the levels of IL-1β in OB supernatants were determined by ELISA. (**J**) The levels of IL-18 were determined by ELISA in OB supernatants. (**K**) The pyroptosis-related proteins caspase-1, IL-1β, IL-18, GSDMD, and cleaved GSDMD in OBs were detected using Western blotting. Statistical analysis was performed using two-tailed Student’s *t*-test. Values are expressed as means ± SEM. *P < 0.05. BMD, bone mineral density; LDH, lactate dehydrogenase; OBs, osteoblasts; OCs, osteoclasts; PbAc, lead acetate.

A co-culture system (0.4 µm) was used to better understand the intercellular communication between PbAc-stimulated OCs and OBs (Figure 5A). OCs were transfected with fluorescent FAM-labeled miR-30a-3p mimics and co-cultured with OBs. Then, the OBs in the lower layer were collected and observed under a fluorescence microscope. A green fluorescent signal was co-localized with the nuclear dye DAPI (Figure 5A), suggesting the successful internalization of FAM-labeled miR-30a-3p mimics. In addition, the levels of miR-30a-3p in OBs were significantly elevated after co-culturing (Figure 5B), indicating that miR-30a-3p was transferred from OCs (upper layer) to OBs (lower layer).

To demonstrate that the communication of miR-30a-3p between cells is dominated by exosomes, we performed an exosome lysis experiment. As shown in Figure 5C, the expression of miR-30a-3p in the Pb-treated OCs was not reduced with the single application of ribonuclease but was significantly decreased with the co-administration of ribonuclease and Triton. This suggests that miR-30a-3p is protected by the bilayer membrane of the exosomes. Furthermore, administration of the exosome synthesis inhibitor GW4869 significantly reduced miR-30a-3p in the supernatant of culture medium from Pb-treated OCs (Figure 5D). In addition, the over-expression of unsealed mir-30a-3p during the co-culture process did not change miR-30a-3p levels in OBs (Figure 5E). These findings indicated that miR-30a-3p was not generated by OBs but was mainly secreted by Pb-treated OCs and was delivered by exosomes.

To investigate the biological effects of Pb-induced OC-Exos miR-30a-3p on OBs, cell viability and the expression of cell death-related proteins in OBs were evaluated after co-culturing with Pb-treated or non-treated OCs. A significant increase in OB death was observed after co-culturing with Pb-treated OCs (Figure 5F), accompanied by elevated release of LDH (Figure 5G) and an increase in PI-positive cells (Figure 5H). These observations suggested substantial membrane damage and cell death. miRNAs have been widely recognized as critical regulators of cell death, including apoptosis, necroptosis, and pyroptosis, by targeting key components of apoptotic and inflammatory pathways [32,33]. Notably, the secretion of the inflammatory cytokines IL-1β (Figure 5I) and IL-18 (Figure 5J) by OBs was significantly enhanced after co-culturing with Pb-treated OCs. Furthermore, the levels of the pyroptosis-related proteins caspase-1, IL-1β, IL-18, GSDMD, and cleaved GSDMD in the Pb-OC-OB group were significantly higher than those in the control group, with *P <* 0.05 (Figure 5K). These results collectively indicate that miR-30a-3p delivered by Pb-treated OC-derived exosomes induces pyroptosis in OBs, leading to their death.

### OC-Exo-miR-30a-3p induced pyroptosis in OBs

**Figure 6:**
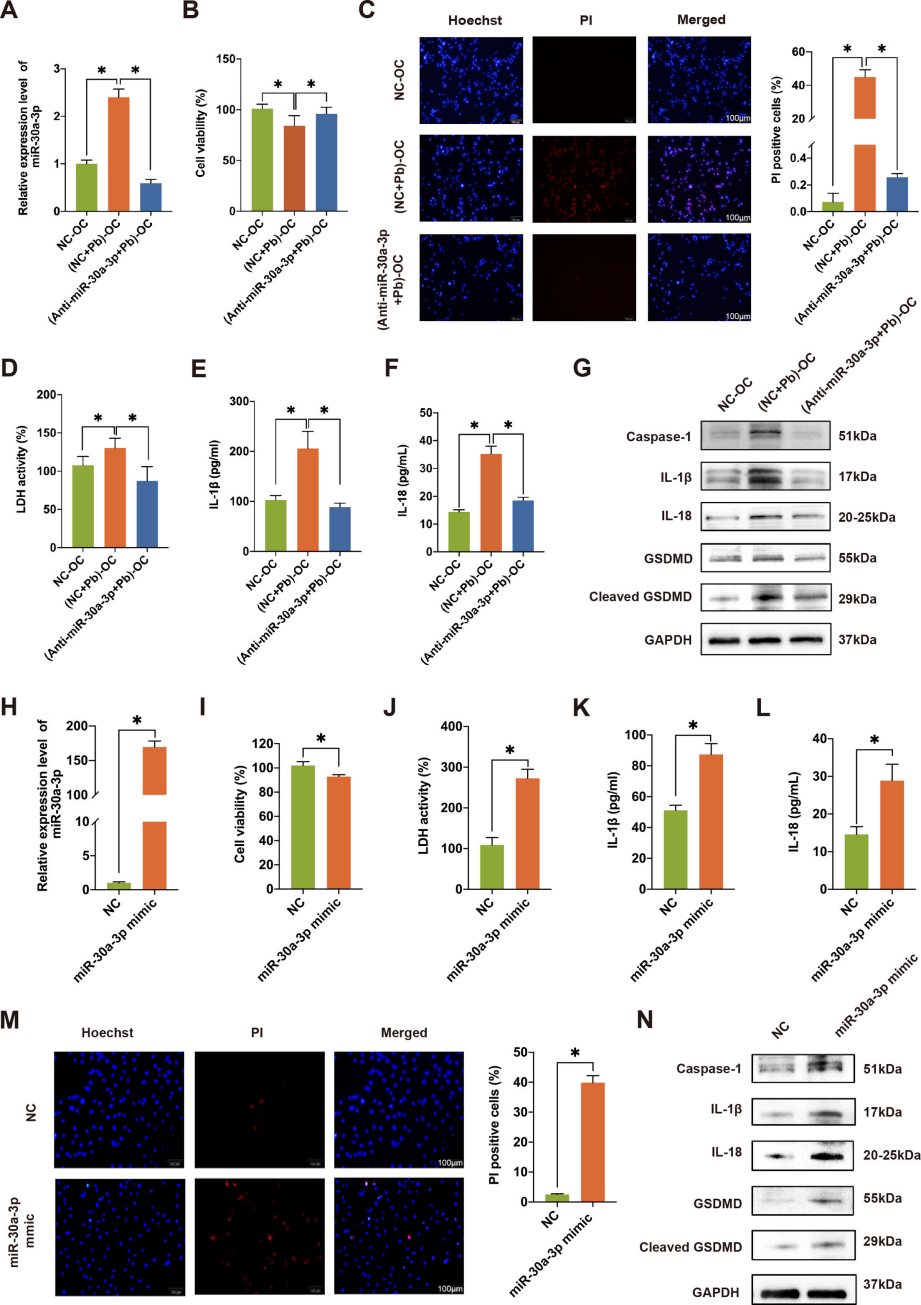
OC-Exo-miR-30a-3p is responsible for the pyroptosis of OBs. OCs were transfected with NC or miR-30a-3p inhibitor and then exposed to Pb and co-cultured with OBs, namely, NC-OC, (NC+Pb)-OC, and (Anti-30a-3*P*+Pb)-OC. The co-culture of OBs with OC transfected miR-30a-3p mimic or non-treatment, namely miR-30a-3p mimic or NC, respectively. (**A**) The level of miR-30a-3p in OBs was determined using RT-qPCR**.** (**B**) The viability of OBs was evaluated using a CCK-8 assay. (**C**) Photomicrographs of double-fluorescent staining with PI (red) and Hoechst 33,342 (blue). Scale bar, 100 μm. (**D**) The LDH release levels of OBs. (**E**) After incubation with (Anti-30a-3*P*+Pb)/NC+Pb/NC-treated OCs for 24 h, the levels of IL-1β were determined by ELISA in the supernatants. (**F**) The levels of IL-18 were determined by ELISA in the supernatants. (**G**) The pyroptosis-related proteins, namely, caspase-1, IL-1β, IL-18, GSDMD, and cleaved GSDMD, in OBs were detected using Western blotting (WB). (**H**) The level of miR-30a-3p in OBs was determined using RT-qPCR. (**I**) The viability of OBs was evaluated using a CCK-8 assay. (**J**) The LDH release of OBs. (**K**) The level of IL-1β was determined by ELISA (**L**) The level of IL-18 was determined by ELISA. (**M**) Images of OBs after double-fluorescent staining with PI (red) and Hoechst 33,342 (blue). Scale bar, 100 μm. (**N**) The pyroptosis-related proteins caspase-1, IL-1β, IL-18, GSDMD, and cleaved GSDMD in OBs were detected using WB. Statistical significance was assessed using two-tailed Student’s *t*-test. Values are means ± SEM. *P < 0.05. BMD, bone mineral density; LDH, lactate dehydrogenase; OBs, osteoblasts; OCs, osteoclasts; PbAc, lead acetate.

To confirm the role of miR-30a-3p in OB pyroptosis, OCs were transfected w/o miR-30a-3p inhibitors and then treated with Pb. PCR results showed that the level of miR-30a-3p in OBs decreased remarkably after co-culturing with (Anti-30a-3*P*+Pb)-treated OCs, verifying the capability of the inhibitor (Figure 6A). The viability of OBs increased significantly when co-cultured with (Anti-30a-3*P*+Pb)-treated OCs (Figure 6B), showing an effective decrease in OB death due to the inhibitor. Consistently, the amount of PI-stained positive cells was also significantly lower than that in the Pb-treated co-culture group (Figure 6C). The level of LDH released from OBs was decreased after co-culturing with (Anti-30a-3*P*+Pb)-treated OCs (Figure 6D). Also, the administration of an Anti-30a-3p agent inhibited the release of the inflammatory factors IL-1β and IL-18 (Figure 6E and F). Elevated expression of the pyroptosis-related proteins caspase-1, IL-1β, IL-18, GSDMD, and cleaved GSDMD in OBs was inhibited by the miR-30a-3p inhibitor (Figure 6G), suggesting that the regulation of OBs in the co-culture system was reliant on miR-30a-3p.

After co-culturing OBs with miR-30a-3p mimic-transfected OCs, the level of miR-30a-3p in OBs was remarkably up-regulated (Figure 6H) and the viability of OBs was significantly decreased (Figure 6I). In addition, LDH levels were increased substantially (Figure 6J), and the expression of the inflammatory factors IL-1β (Figure 6K) and IL-18 (Figure 6L) and the amount of positive PI-stained cells (Figure 6M) were also significantly increased. Also, the expression of pyroptosis-related proteins including caspase-1, IL-1β, IL-18, GSDMD, and cleaved GSDMD was significantly elevated (Figure 6N). The above results revealed that pyroptosis in OBs induced by OC-Exo-miR-30a-3p contributed to their death.

### OC-Exo-miR-30a-3p induced the pyroptosis of OBs via an NF-kB-dependent pathway

**Figure 7:**
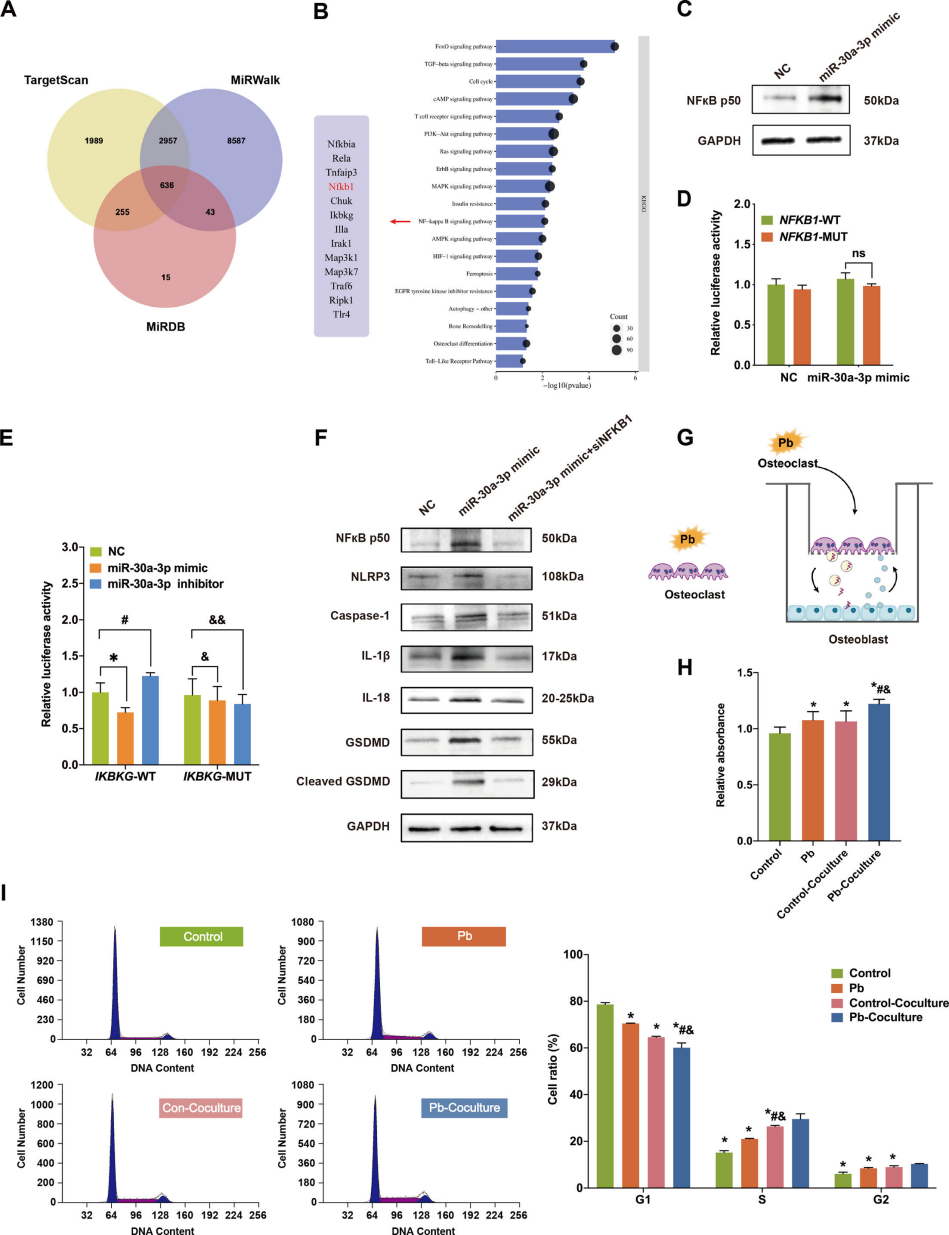
OC-Exo-miR-30a-3p induces pyroptosis in osteoblasts (OBs) via an NF-κB-dependent pathway, and this pyroptosis induction in OBs also enhances the proliferation of osteoclasts (OCs) in a positive-feedback manner. (**A**) The potential target genes containing the miR-30a-3p seed sequence were predicted as the overlapping results from the TargetScan, MiRwalk, and MiRDB databases. (**B**) KEGG pathway enrichment analysis of the 636 target genes. (**C**) The expression of NF-κB1-encoded protein in NC/miR-30a-3p mimic-treated OBs was measured using WB. (**D**) The binding affinity of miR-30a-3p to NF-κB1 was assessed using a dual-luciferase assay. (**E**) The regulatory effects of IKBKG on miR-30a-3p were detected by dual luciferase assay. (**F**) The levels of pyroptosis-related proteins in OBs in NC, miR-30a-3p mimic, or with NF-κB1 knockdown were measured using WB. (**G**) Schematic illustration of non/Pb-treated OCs incubated with OBs or exposed to Pb directly. OCs cultured with OBs were classified as control–co-culture group (without PB pre-treatment) or Pb-co-culture group (with Pb pre-treatment). (**H**) The cell proliferation was evaluated using a CCK-8 assay. (**I**) The cell cycle of OCs was analyzed using flow cytometry. Statistical significance was analyzed using two-tailed Student’s *t*-test. Values are means ± SEM. *Compared with the Control group; #Compared with the Pb group; &Compared with the control–co-culture group, *P* < 0.05.

To further explore the regulatory mechanism of miR-30a-3p in OB pyroptosis, the TargetScan, MiRwalk, and MiRDB databases were utilized to screen for potential targets of miR-30a-3p (Figure 7A). Subsequently, KEGG pathway enrichment analysis was performed, and the results indicated that miR-30a-3p may be involved in a variety of biological processes (Figure 7B). Interestingly, the NF-κB signaling pathway was previously found to be associated with the activation of the inflammasome NLRP3 in the classical pyroptosis pathway [34,35]. Thus, the protein level of NF-κB1 was measured as a candidate downstream target in the pyroptosis of OBs. The results demonstrated a significant increase in NF-κB p50 levels in OBs following co-culture with OCs transfected with miR-30a-3p mimics (Figure 7C). This finding suggests that OC-Exo-miR-30a-3p exerts a positive regulatory effect on the target gene. However, the dual luciferase reporter assay revealed no significant alteration in luciferase activity when NF-κB1 3'-UTR and miR-30a-3p were co-expressed (Figure 7D), indicating the absence of a direct targeting effect between NF-κB1 3'-UTR and miR-30a-3p. Alternatively, miR-30a-3p may indirectly activate NF-κB 1. Consequently, our attention shifted toward IKBKG, which is also enriched in the NF-κB 1 signaling pathway and exerts a negative regulatory influence on the NF-κB signaling pathway. The results showed that the relative luciferase activity of miR-30a-3p mimics in the transfection group was significantly lower than that in the normal control group (*t* = 3.065, *P* = 0.023). In addition, luciferase activity in the miR-30a-3p-inhibitor group was significantly higher than that in the control group (*t* = −2.857, *P* = 0.046). In the mutant IKBKG group, luciferase activity in the miR-30a-3p-mimic and miR-30a-3p-inhibitor groups was not significantly different from that in the control group (*t* = 0.486, *P* = 0.685; *t* = 0. 835, *P* = 0. 451) (Figure 7E), indicating that miR-30a-3p directly binds to the 3'UTR of the IKBKG gene and plays a regulatory role. Although OC-Exo-miR-30a-3p was still present, the expression of NLRP3 and pyroposis-related proteins was decreased significantly following NF-κB1 knockdown (Figure 7F). Collectively, these findings suggest that miR-30a-3p promotes OB pyroptosis by directly targeting IKBKG and indirectly activating the NF-κB signaling pathway, providing new insights into the molecular mechanisms underlying bone-related diseases.

### OC-Exo-miR-30a-3p induced pyroptosis in OBs and promoted OC proliferation in a positive-feedback loop

It has been reported that the pro-inflammatory factors IL-1β and IL-18 secreted by pyroptotic cells can regulate OC proliferation [35,36]. Therefore, OC proliferation was measured after pyroptosis induction in OBs by OC-Exo-miR-30a-3p. The OCs with Pb-treatment alone were established as a control (Pb group) to eliminate the direct effect of Pb on the proliferation of OCs. The OBs were co-cultured with OCs w/o Pb exposure; the Pb-co-culture or con-co-culture groups are depicted in Figure 7G. In addition, flow cytometry demonstrated a decreased ratio of G1-phase cells and an increase in S-phase cells in the Pb-co-culture group compared with Pb treatment alone and the con-co-culture group (Figure 7I). This indicated that OB pyroptosis promoted the transition of OCs from the G1 phase to S phase to stimulate proliferation. These results suggest that Pb exposure may trigger a positive-feedback loop between OCs and OBs. OC-derived exosomal miR-30a-3p induces the release of pro-inflammatory factors (such as IL-1β and IL-18) from OBs via OB pyroptosis, subsequently promoting OC proliferation.

## Discussion

**Scheme 1:**
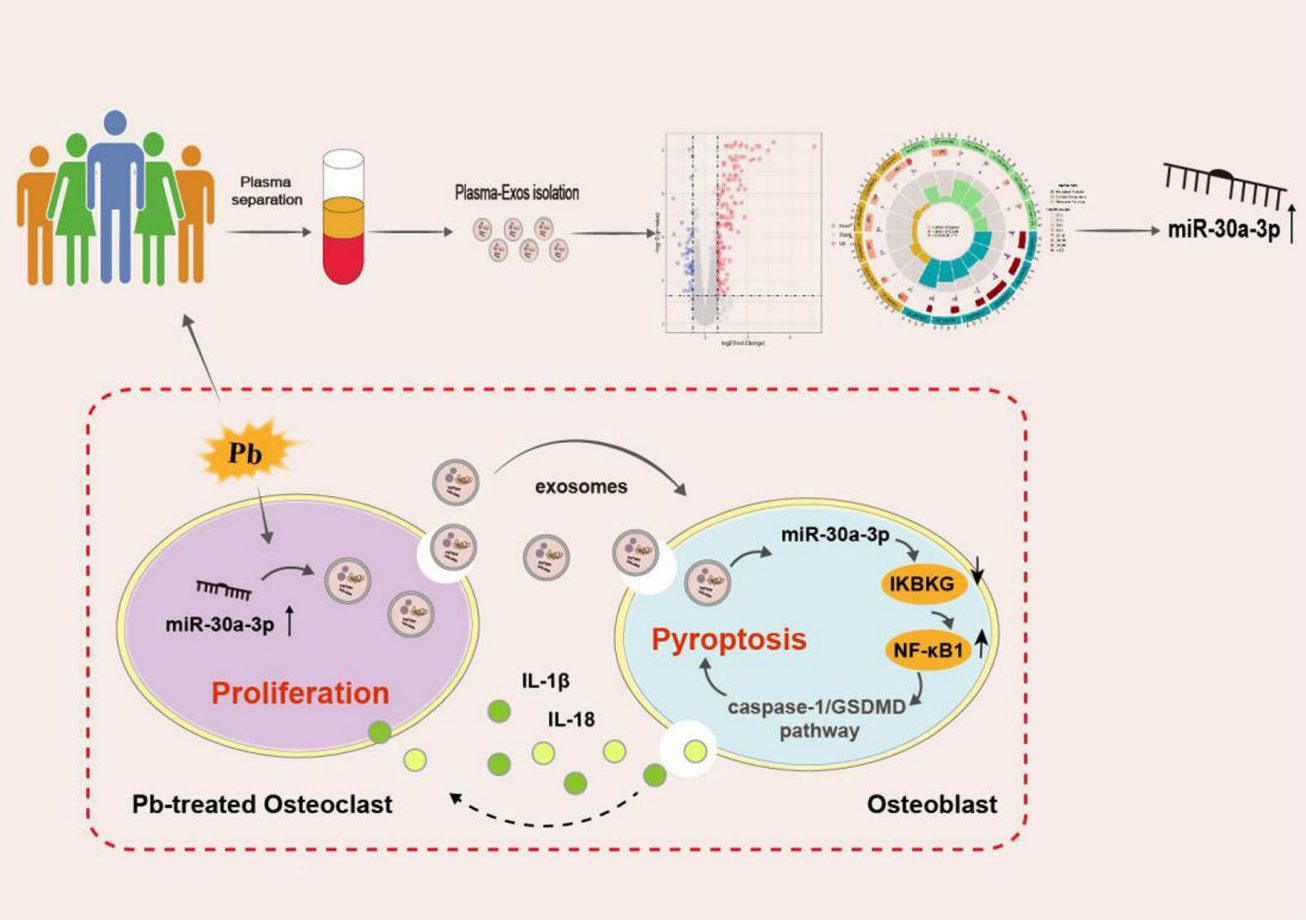
The expression of exosome miR-30a-3p was up-regulated in the lead (Pb)-exposed group, suggesting that the abnormal expression of exosome miR-30a-3p derived from osteoclasts may be involved in the Pb-exposed group. After Pb treatment, miR-30a-3p was up-regulated in OCs. In cell-to-cell communication, Exos-packaged miR-3 0 a-3p secreted from OCs regulates the expression of NF-κβ1 and activates the caspase-1/GSDMD signaling pathway in OBs stimulating pyroptosis. Meanwhile, the inflammasomes IL-1β and IL-18 released from OBs promote the proliferation of OCs. Thus, the homeostasis between bone resorption in OCs and bone formation in OBs is destroyed, which affects the bone remodeling process and induces osteoporosis.

Chronic Pb exposure due to environmental and occupational contact is recognized as a serious threat to skeletal integrity [37,38]. Bones are the main tissue for Pb accumulation and are frequently a site of Pb toxicity that results in typical clinical outcomes such as osteoporosis and bone fracture. In diseases induced by Pb exposure, the removal of Pb^2+^ is necessary to ensure treatment efficacy. However, the measurement of BLLs is not routinely included in the regular diagnosis of osteoporosis, which may lead to misdiagnose of Pb-induced osteoporosis. As summarized in Scheme 1, our study demonstrated that elevated BLLs in individuals after chronic Pb exposure were commonly associated with lower BMD and osteoporosis, which is consistent with previous studies [39,40]. We also found that OC-Exos were enriched in the plasma of low-BMD patients with Pb exposure. Subsequent results demonstrated that OC-Exo-packaged miR-30a-3p was expressed at a high level in plasma samples from patients and Pb-induced osteoporotic rats. Further, *in vivo* and *in vitro* studies confirmed that OC-Exo-packaged miR-30a-3p was transferred to OBs and inhibited bone formation, possibly via targeting IKBKG to initiate caspase-1 activation and the secretion of IL-1β and IL-18, thus accelerating the progression of osteoporosis. Meanwhile, this interaction between OCs and OBs could be suppressed by the administration of miR-30a-3p inhibitor as a prevention for osteoporosis.

Exosomes possess the smaller structure, high secretion rate, and the ability to carry a substantial amount of genetic information, making them a promising biomarker in disease diagnosis, prognosis, and monitoring treatment response [41]. In our study, exosome levels were significantly increased in the low-BMD Pb-exposed population, suggesting the participation of exosomes and their content in the occurrence and development of this pathological condition. Thus, illustrating the specific expression of exosomes and their content would facilitate our understanding and detection of Pb-induced osteoporosis caused by environmental pollution. As the most common content in exosomes, Exo-miRNA has been widely studied due to its stable expression, its presence in various species, and its mature function compared with non-coding RNAs. Its key role in bone homeostasis and remodeling has been broadly studied [25,42]. Therefore, we studied the expression and characterization of Exo-miRNA as a biomarker of Pb-induced osteoporosis. This is the first report showing that Exo-miR-30a-3p was overexpressed in Pb-exposed individuals with low-BMD compared with a healthy population, a low-BMD population without Pb exposure, and a normal-BMD population with Pb exposure. This study also showed that the sensitivity and specificity of miR-30a-3p expression in peripheral blood for the diagnosis of Pb-induced osteoporosis were 93.9% and 84.6%, respectively. The expression of miR-30a-3p in the peripheral blood exhibited significant diagnostic efficiency for Pb-induced osteoporosis, providing a new strategy and target for the prevention and treatment of Pb-related bone disease. The expression of osteoclastic markers (Sema4D and TRAcP5) was detected, indicating that these exosomes were derived from OCs. This finding was further validated in a Pb-induced rat model of osteoporosis. Collectively, these results suggest that circulating OC-Exo-packaged miR-30a-3p may serve as a specific biomarker for Pb-induced osteoporosis. Compared with traditional indicators such as BLLs and BMD, miR-30a-3p offers distinct advantages in detecting early-stage molecular-level dysregulation in bone metabolism induced by Pb toxicity. While BLLs primarily reflect cumulative exposure, BMD lacks sensitivity in early disease stages and cannot differentiate Pb-induced bone damage from other forms of osteoporosis. In contrast, miR-30a-3p represents a non-invasive and highly sensitive diagnostic tool.

The current concept of Pb-induced osteoporosis can be summarized as the competitive binding of Pb^2+^ to Ca^2+^-signaling proteins [43], where the native metal-binding site geometry of Pb either activates host proteins or induces malfunction to modulate the replacement of Ca^2+^ [44]. However, the direct or indirect effects of Pb on bone cell differentiation and function are merely hypothesized [45]. Recent studies have reported decreased viability of OBs in bone homeostasis following Pb exposure [2,46]. Our study further confirmed that Pb disturbed homeostasis and activated the remodeling process in bone. We also showed that OC-derived exosomal miR-30a-3p was detected in low-BMD patients with Pb exposure. Also, *in vitro* studies demonstrated that OC-Exo-miR-30a-3p specifically entered OBs and induced pyroptosis, severely reducing the amount of OBs. These results suggested that Pb can disturb the balance between OBs and OCs, thus inducing osteoporosis.

The relationship between OBs and OCs has been extensively studied and multiple types of cellular interactions have been identified. Direct interaction through the OPG/RANKL/RANK, RANKL/LGR4/RANK, EFNB2-EPHB4, FASL-FAS, or SEMA3A-NRP1 pathways has been shown to regulate differentiation and apoptosis [16]. OBs secrete different molecules such as M-CSF, RANKL/OPG, WNT5A, and WNT16 to regulate the differentiation of OCs. Conversely, OCs also influence bone formation induced by OBs through secretion of soluble factors including S1P, SEMA4D, CTHRC1, and C3 [16]. Recent studies have reported that exosomes may be a novel mediator of osteoporosis. Specifically, exosomal miRNAs such as miR-214–3p have been identified as messengers for cell–cell communication. In a distinct osteoporosis model using ovariectomized mice, it was demonstrated that OC-derived exosomal miR-214–3p disrupts communication between OCs and OBs, thereby inhibiting bone formation [23]. However, the identification of different disease pathogeneses through the fingerprinting of exosomal miRNAs and distinguishing osteoporosis with the same clinical characteristics have not been achieved. Our results show that Pb exposure can specifically up-regulate both the expression of exosomes and OC-Exo-miR-30a-3p. These results support the hypothesis that osteoporosis specifically induced by Pb exposure may follow a unique pathogenesis, distinct from other forms of the disease. Our findings underscore the potential of exosomal miRNAs as promising biomarkers and therapeutic targets in osteoporosis.

Our study revealed that Pb may affect OB function not only by reducing the viability of OBs but also by secreting exosomal miR-30a-3p derived from OCs to regulate OBs. Additionally, miR-30a-3p was only up-regulated in OBs after co-culturing with Pb-treated OCs, and it was not increased following direct Pb treatment. These results suggested that Pb treatment alone did not induce miR-30a-3p secretion by OBs and that functional miR-30a-3p was mainly secreted by OCs. Furthermore, our results indicated that the exosome was the predominant means for the transfer of miR-30a-3p from OCs to OBs after Pb exposure, as miR-30a-3p was undetectable after the administration of a membrane-dissolving agent. In addition, the bone-targeting property of OC-Exo-miR-30a-3p was demonstrated *in vivo*, visualized as an intensive fluorescent signal in the femurs of mice. High uptake in the liver, kidney, and spleen was consistent with the results reported by Lu and Zhang et al., indicating the wide circulation of miRNAs [23].

Bone is a vital site for pyroptosis [47]. In this study, we found that pyroptosis occurred in OBs from Pb-treated rats. Related studies have shown that the miR-30a family functions as an important regulator of pyroptosis [27,48]. Thus, we extensively studied the cause and effect relationship between miR-30a-3p and pyroptosis in OBs. Pyroptosis is a form of programmed cell death that was first discovered by Zychlinsky et al.[49]. The key gene in the pyroptosis signaling pathway, NOD-like receptor thermal protein domain associated protein 3 (NLRP3) inflammasome, is an intracellular multiprotein complex that regulates the maturation and secretion of caspase-1-dependent proinflammatory cytokines such as IL-1β and IL-18, mediates inflammation, and induces pyroptosis [34]. In our results, a positive correlation was demonstrated between the increase in miR-30a-3p and activation of NLRP3 and its downstream genes, including IL-1β and IL-18 [50]. Further, miR-30a-3p inhibitor treatment reversed this effect. Luciferase assay revealed that miR-30a-3p did not regulate the transcriptional activity of NLRP3 3′UTR. The above results show that the presence of an alternative mode or indirect regulation between miR-30a-3p and NLRP3.IKBKG, also known as NF-κB essential modulator (NEMO), is a key to the negative regulation of NF-κB signaling pathway. Under normal conditions, IKBKG binds to IκB proteins, preventing the nuclear translocation of NF-κB transcription factors. When cells are stimulated, IKBKG is phosphorylated, leading to its dissociation from IκB proteins and release of NF-κB transcription factors. Therefore, down-regulation or inactivation of IKBKG promotes activation of the NF-κB signaling pathway, enhancing inflammatory responses and immune reactions [51]. Dual-luciferase reporter assays showed that miR-30a-3p directly bound to the 3'UTR of the IKBKG gene to play a regulatory role. These findings indicate that miR-30a-3p negatively regulated IKBKG, leading to increased expression of NF-κB1, activation of the NLRP3 inflammasome, and up-regulation of a series of proteins related to pyroptosis. Ultimately, this cascade of events induces pyroptosis in OBs.

This study has some limitations. First, the effect of OC-Exo-miR-30a-3p on other cells that may contribute to this disease, such as bone marrow mesenchymal stem cells, was not investigated. Second, the use of miR-30a-3p inhibitors as an effective therapy for Pb-induced osteoporosis was not carried out, but this would be the focus of future research. Currently, the optimization of injected dosages and delivery methods for inhibitors has not been explored. The further study of these gaps would assist in the comprehensive understanding of pyroptosis signaling pathways in bone due to Pb exposure.

## Conclusion

miR-30a-3p is highly expressed in exosomes derived from OCs and mediates OB pyroptosis, inhibiting bone formation through cellular communication in Pb-induced osteoporosis. Therefore, OC-Exo-packaged miR-30a-3p may be a novel risk factor of Pb-induced osteoporosis and holds prognostic value in evaluating bone formation.

**Clinical Perspectives**

- Lead (Pb) exposure contributes to the development of low bone mineral density and osteoporosis through its chronic accumulation in bone tissue, disrupting normal bone metabolism.
- Osteoclast-derived exosomes enriched with miR-30a-3p are significantly associated with Pb-induced bone loss, driving pyroptosis in osteoblasts via the NLRP3 inflammasome pathway.
- Targeting miR-30a-3p offers a promising biomarker and therapeutic strategy to diagnose and manage Pb-induced osteoporosis, enhancing clinical interventions for patients with environmental Pb exposure.

## Data Availability

All data supporting the findings of this study are included in the manuscript and its supplementary materials. Human sequencing data have been deposited in the NCBI BioSample database under BioProject accession PRJNA1061492. Similarly, cellular sequencing data have been uploaded to the NCBI BioSample database under BioProject accession PRJNA1061766. These datasets can be accessed through the following links: https://www.ncbi.nlm.nih.gov/bioproject/?term=PRJNA1061492 https://www.ncbi.nlm.nih.gov/bioproject/?term=PRJNA1061766 Predictive analyses for target genes of miR-30a-3p were conducted using the online databases TargetScan (www.targetscan.org) and miRDB (www.mirdb.org/miRDB). For any additional data or materials not provided in the manuscript or supplementary files, inquiries can be directed to the corresponding author via email at sosolou@jscdc.cn.

## Open Access

This article has been published open access under our Subscribe to Open programme, made possible through the support of our subscribing institutions, learn more here: https://portlandpress.com/pages/open_access_options_and_prices#conditional

## Abbreviations

ALP: alkaline phosphatase
BLL: blood lead level
BMD: bone mineral density
CTSK: cathepsin K
Exos: exosomes
IL-1: interleukin-1
IL-8: interleukin-8
LDH: lactate dehydrogenase
M-SCF: recombinant mouse SCF protein
Micro-CT: micro-computed tomography
NF-κB: permissible concentration-time weighted average
NLRP3: NOD-like receptor thermal protein domain associated protein 3
NTA: nanoparticle tracking analysis
OBs: osteoblasts
OC-Exos: osteoclast-derived exosomes
OCs: osteoclasts
OP: osteoporosis
PbAc: lead acetate
RANKL: recombinant mouse TRANCE/RANKL 11 protein
RAW264.7: mouse mononuclear macrophage leukemia cells
Sema4D: semaphorin 4D
TEM: transmission electron microscopy
TRAP: tartrate-resistant acid phosphatase
TRAcP5: tartrate-resistant acid phosphatase 5b
qRT-PCR: real-time quantitative reverse transcription PCR
